# Circulating levels of copeptin predict outcome in patients with pulmonary arterial hypertension

**DOI:** 10.1186/1465-9921-14-130

**Published:** 2013-11-19

**Authors:** Nils P Nickel, Ralf Lichtinghagen, Heiko Golpon, Karen M Olsson, Korbinian Brand, Tobias Welte, Marius M Hoeper

**Affiliations:** 1 Department of Respiratory Medicine, Hannover Medical School, 30623 Hannover, Germany; 2 German Center of Lung Research, Hannover, Germany; 3 Institute for Clinical Chemistry Hannover Medical School, Hannover, Germany

## Abstract

**Objective:**

To determine the levels of circulating copeptin in patients with pulmonary arterial hypertension (PAH), and to evaluate its relation with disease severity, outcome and response to treatment.

**Background:**

Vasopressin is a key regulator of body fluid homeostasis. The co-secreted protein copeptin serves as surrogate for plasma vasopressin levels and increases in acute and chronic left ventricular dysfunction. Copeptin has not been studied in PAH.

**Methods:**

Serum copeptin levels were evaluated in a retrospective cohort of 92 treatment-naïve patients with PAH, 39 patients with normal right ventricular hemodynamics (diseased controls) and 14 apparently healthy individuals (healthy controls). In a second prospective cohort of 15 patients with PAH, serial changes of copeptin levels after initiation of PAH treatment were measured. Copeptin levels were compared with clinical, biochemical and hemodynamic parameters as well as response to treatment and clinical outcome.

**Results:**

Circulating copeptin levels were elevated in PAH patients compared to diseased controls (20.1 pmol/l vs. 5.1 pmol/l; p = 0.001). Baseline levels of copeptin correlated with NYHA functional class (r = 0.46; p = 0.01), 6 minute walking distance (r = -0.26; p = 0.04), NT-proBNP (r = 0.49, p = 0.01), creatinine (r = 0.39, p = 0.01) and estimated glomerular filtration rate (r = -0.32, p = 0.01). Copeptin levels did not correlate with hemodynamics but decreased after initiation of PAH therapy (p = 0.001). Elevated copeptin levels were associated with shorter survival (p < 0.001) and independent predictors of mortality in a multiple Cox regression analysis (HR1.4; 95% confidence interval 1.1-2.0; p = 0.02).

**Conclusions:**

Patients with PAH had elevated copeptin levels. High circulating levels of copeptin were independent predictors of poor outcome, which makes copeptin a potentially useful biomarker in PAH.

## Introduction

Pulmonary arterial hypertension (PAH) is a disease characterized by progressive remodeling of the pulmonary vasculature, leading to right ventricular strain and subsequent right heart failure [1]. Several clinical, biochemical and hemodynamic parameters are associated with outcome [2] but survival in patients with PAH is mainly dependent on preserved right ventricular function [3].

Common clinical symptoms of advanced right heart failure are lower extremity edema resulting from volume overload and disturbed body fluid homeostasis [3]. The regulation of body fluid homeostasis in chronic heart failure is complex and related to cardiac output and peripheral arterial resistance [4].

To compensate for a decrease in effective circulatory volume, the activation of neurohumoral reflexes mediated by the sympathetic nervous system leads to activation of the renin-angiotensin-aldosterone system, and the nonosmotic release of arginine vasopressin (AVP), all of which result in sodium or water retention [5].

The levels of circulating AVP underlie a complex feed forward and feedback regulation. Among the stimuli that lead to AVP release are increased plasma osmolality, decreased arterial pressure, reduced cardiac filling and neurohumoral peptides such as angiotensin [6].

Once released into the circulation, AVP exerts is peripheral effects by binding to tissue specific receptors. The two predominant receptors for AVP are the vasopressin 1(V_1_) and the vasopressin 2(V_2_) receptor. The V_1_ receptor mediates arteriolar vasoconstriction and the V_2_ receptor mediates water reabsorption via induction of aquaporins in the collecting ducts of the kidney [7].

AVP has a short plasma half-life and is unstable in isolated plasma [8]. In addition to that, 90% of the circulating AVP protein is bound to platelets, resulting in varying AVP levels, depending on sampling handling and storage.^9,10^ The small molecular size of the AVP protein makes it not suitable for conventional sandwich immunoassays [9]. For all of these reasons, AVP measurements have never become clinical routine.

AVP derives from a precursor protein, pre-pro-vasopressin, which consists of a signal peptide, AVP, neurophysin II, and copeptin [10]. These three peptides are stochiometrically secreted from the posterior pituitary [11]. Thus, copeptin levels can be used as a surrogate of AVP release [9,12]. Copeptin is a well-established surrogate marker for AVP-release that is stable *ex vivo* in serum and plasma and therefore suitable for retrospective analyses [9].

Copeptin has been shown to be of prognostic importance in a variety of cardiovascular pathologies [13-15]. In chronic left heart failure, increased levels of copeptin were correlated with hyponatremia and independently linked to excess mortality [14].

Compared to left heart disease the neurohumoral axis is less extensively studied in patients with pulmonary hypertension and right-sided cardiac dysfunction. Patients with PAH show increased sympathetic nervous system activity, elevated levels of endothelin, norepinephrine [16] renin and aldosterone [17,18] and it is likely that the AVP system is also activated. Of note, elevated plasma volume and low serum sodium concentrations are associated with excess mortality in PAH patients [19,20].

In the present study we studied copeptin levels in patients with PAH and the potential role of copeptin as biomarker in this disease.

## Patients and controls

We included 107 patients with PAH (Group 1 Dana Point classification) in this study. The first cohort was studied retrospectively and consisted of 92, treatment-naïve patients with NYHA functional class II to IV referred to Hannover Medical School between 2002 and 2010.

The second patient cohort was independent from the first cohort and consisted of 15 consecutive patients who were prospectively studied after they had been diagnosed with PAH. The PAH targeted therapy for the second cohort is shown in Additional file 1: Table S2.

In all patients, the diagnosis of PAH was based on standard criteria with confirmation by right heart catheterization and exclusion of other forms of pulmonary hypertension by echocardiography, pulmonary function testing, chest X-ray, ventilation–perfusion scanning, chest computed tomography angiography and laboratory studies [3].

The first control group (healthy controls) consisted of 14 apparently healthy individuals with no medical history and who were on no medication. The second control group consisted of 39 age- and gender-matched patients who were referred to our clinic for assessment of pulmonary hypertension by right heart catheterization, based on the suspicion of PAH by previous functional heart evaluation. In all patients the diagnosis of PH/PAH (defined by mPAP > 25 mmHg, PCWP < 15 mmHg) was ruled out.

Baseline blood samples for the PAH cohorts and the diseased controls cohort were collected at the time of the initial right heart catheterization before the initiation of any PAH-targeted therapy. In the second PAH cohort, an additional blood sample was obtained during follow up right heart catheterization after 3 to 6 months. All blood samples were immediately cooled on ice and centrifuged at 4°C. Serum samples were divided into aliquots and stored frozen at -80°C.

All patients and control individuals gave written, informed consent for storage of serum samples and future biomarker analyses at the time when the samples were obtained, an approach that was approved by the institutional review boards of Medical School Hannover (Nr. 3558).

### Biochemical measurements

Quantitative determination of copeptin (CT-proAVP) values from serum samples was performed using an immunoluminometric assay (*Brahms CT-proAVP* LIA, Brahms GmbH, Hennigsdorf, Germany) on the tube luminometer *Lumat LB 9507* (Berthold Technologies GmbH, Bad Wildbad, Germany). The functional sensitivity of the assay was shown to be <1.0 pmol/l, an interassay CV <10% was found for copeptin concentrations >2.5 pmol/l. NT-proBNP serum values were quantified using an electrochemiluminiscence (ECLIA) assay on the *Modular E170* analyser (Roche Diagnostics, Mannheim, Germany). Serum creatinine was determined enzymatically on the Modular P800 (Roche Diagnostics), and sodium values were quantified on the *ISE900* by indirect potentiometry (Roche Diagnostics).

### Statistical analysis

Variables are presented as absolute numbers, percentage or median (interquartile range). Differences in demographic, clinical, biochemical and hemodynamic variables between PAH-patients and non-PAH-patients were assessed using Spearman rank correlation for continuous variables, χ^2^ test for nominal variables, and NYHA class. Differences in copeptin levels among the healthy and diseased controls and the first PAH cohort and PAH-subgroups were assessed using Kruskal–Wallis one-way analysis of variance. Differences among patients with copeptin levels below or above 13.2 pmol/l were calculated by using Mann–Whitney U-test for continuous, and χ^2^-test for categorical variables.

Relations between copeptin level and baseline variables were assessed by Spearman rank correlation coefficients. The Wilcoxon signed rank test was used to compare changes in variables after initiation of treatment.

For survival analysis death or lung transplantation was defined a priori as the primary composite endpoint. For Cox regression analysis variables were categorized as indicated in Table 1. Simple Cox regression analyses were performed to identify predictors of death. For multiple stepwise forward Cox regression, copeptin was tested with biochemical variables (N-terminal prohormone of brain natriuretic peptide, creatinine, estimated glomerular filtration rate calculated according the Chronic Kidney Disease Epidemiology Collaboration formula (eGFR-CKD-EPI), serum sodium concentration). For this analysis, variables with a p-value of less then 0.05 were entered and variables with a p-value of greater than 0.1 were removed.

**Table 1:** Risk of death in relation to demographic, clinical, hemodynamic and biochemical baseline assessment

**A: Single parameter cox regression analysis for clinical, biochemical and hemodynamic parameters, including copeptin**		
	**Single model**	
**Parameter**	**HR (CI)**	**p-value**
Age (per 5 years↑)	1.0 (0.9 – 1.1)	0.464
Gender	1.3 (0.6 – 2.5)	0.521
6MWD (30 m↓)	1.3 (1.1- 15)	0.001
NYHA	2.4 (1.2 –3.4)	0.012
Edema	1.0 (0.5 – 1.2)	0.835
S-Na (1 mmol/l ↓)	1.1 (1.1 – 1.2)	0.023
Creatinine (per 10 μg/l ↑)	1.0 (1.0 – 1.2)	0.015
eGFR (CKD-EPI) (per 10 ml/min ↓)	1.4 (1.2 – 2.2)	0.013
NT-proBNP (per 100 pg/ml ↑)	1.2 (1.1 -1.5)	0.019
Copeptin (per 5 pmol/l ↑)	1.9 (1.4 – 2.4)	0.001
RA (per 1 mmHg ↑)	1.2 (1.0 – 1.4)	0.040
mPAP (per 5 mmHg ↑)	1.0 (0.8 – 1.4)	0.081
CI (0.5 ml/min/m^2^ ↓)	1.3 (1.2 – 1.6)	0.018
PVR (50 dyn · sec · cm^-5^ ↑)	1.1 (0.9 – 1.2)	0.105
SvO_2_ (-5% ↓)	1.3 (1.1 – 1.3)	0.017
**B: Stepwise forward cox regression analysis for biochemical variables including copeptin**		
	**Stepwise forward model for clinical variables including copeptin**	
**Parameter**	**HR (CI)**	**p-value**
S-Na (1 mmol/l ↓)	1.0 (0.9 – 1.2)	0.796
Creatinine (per 10 μg/l ↑)	0.7 (0.5 – 1.1)	0.819
eGFR (CKD-EPI) (per 10 ml/min ↓)	0.9 (0.9 – 1.0)	0.129
NT-proBNP (per 100 pg/ml ↑)	0.9 (0.8 – 1.2)	0.581
Copeptin (per 5 pmol/l ↑)	1.4 (1.1 -2.0)	0.015

Data are from the retrospective cohort (cohort 1).

Estimated HRs, 95% CIs, and p values were calculated by simple and forward Cox regression analyses.

Definition of *abbreviations*: *6MWD* 6 minute walking distance, *NYHA* New York Heart Association, *S-Na* Serum Sodium concentration, *eGFR (CKD-EPI)* estimated glomerular filtration calculated according the Chronic Kidney Disease Epidemiology Collaboration formula, *NT-proBNP* N-terminal prohormone of brain natriuretic peptide, *RA* right atrial pressure, *mPAP* mean pulmonary artery pressure, *CI* cardiac index, *PVR* pulmonary vascular resistance, *SvO*_2_ mixed venous oxygen saturation.

Receiver operating characteristics curves (ROC) of NT-proBNP and copeptin were generated and c-statistics were calculated to obtain the NT-proBNP and copeptin cutoff with the highest sensitivity and specificity to predict the risk of death or transplantation after 3 years in cohort 1.

Kaplan Meier curves were generated to estimate and illustrate survival in patients.

Statistical significance between groups were assessed using the Log-Rank (Mantel Cox) method.

P-values < 0.05 were considered statistically significant.

## Results

### Patient’s characteristics

The first (retrospective) patient cohort consisted of 92 patients (66% female) with a median age of 57 (IQR 48–69) years. Median follow up was 55 (IQR 39–72) months. Of the 92 patients, 27 died (29%) and 3 underwent lung transplantation (3%).

The second (prospective) cohort consisted of 15 patients (62% female) with a median age of 58 (IQR 49–65) years. No patient was lost to follow up.

The diseased control group consisted of 39 patients (68% female) with a median age of 53 (IQR 37–71) years. There were no significant differences in age or gender between the PAH cohorts and the diseased control group (Table 2). The final diagnoses of the diseased controls were: chronic obstructive pulmonary disease (30%), sleep apnea (5%), valvular heart disease (8%), interstitial lung disease (23%), connective tissue disease (34%). In patients with CTD the diagnoses related to dyspnea were non-specific interstitial pneumonia, pleural effusion, atelectasis, respiratory muscle weakness and pericardial effusion.

**Table 2:** Baseline characteristic Of PAH-Patients and controls

	**PAH cohort 1 (n = 92)**	**PAH cohort 2 (n = 15)**	**Diseased controls (n = 39)**	**Healthy controls (n = 14)**
Age (years)	57 (48–69)	58 (49–65)	53 (37–71)	39 (27–53)
Gender (female%)	66	62	68	29
6MWD [m]	335 (248–427)	352 (295–413)	389 (280–510)	
NYHA class [%]				
I	0	0	40	
II	24	20	42	
III	71	67	18	
IV	5	13	0	
Edema [%]	39	45	14	
S-Na [mmol/l]	139 (137–142)	140 (138–141)	142 (139–144)	
Creatinine [μg/l]	83 (67–95)	85 (73–98)	79 (67–93)	
eGFR (CKD-EPI) [ml/min]	83 (68–98)	103 (79–99)	89.4 (72–118)	
NT-proBNP [pg/ml]	2125 (588–3225)	2063 (990–2466)	474 (80–698)	
Copeptin [pmol/l]	20.1 (7–25)	24.5 (8–37)	5.1 (2–7)	1.8 (1.4 -2.2)
RA [mmHg]	6.1 (4–9)	7.7 (5–8)	1.9 (2.5)	
PCWP [mmHg]	8.6 (5–11)	7.9 (4–9)	7.8 (3.0)	
mPAP [mmHg]	46 (37–54)	49 (43–56)	17.6 (12–23)	
CI [ml/min/m^2^]	2.4 (1.8-2.8)	1.9 (1.6-2.5)	2.6 (2.3-2.8)	
PVR [dyn · sec · cm^-5^]	792 (469–1024)	1004 (696–1325)	191 (120–271)	
SvO_2_ [%]	63.1 (57–70)	61.2 (56–68)	72 (67–77)	
Diagnosis [%]				
IPAH	64	63	-	
PAH associated with Congenital heart disease	11	7	-	
PAH associated with Connective tissue disease	25	30	-	

Shown are baseline data from PAH cohort 1, cohort 2 as well as diseased and healthy controls.

Except for sex, NYHA class, edema and diagnosis (%), data are shown as median (interquartile range, IQR).

Definition of *abbreviations*: *6MWD* 6 minute walking distance, *NYHA* New York Heart Association, *S-Na* Serum Sodium concentration, *eGFR (CKD-EPI)* estimated glomerular filtration calculated according the Chronic Kidney Disease Epidemiology Collaboration formula, *NT-proBNP* N-terminal prohormone of brain natriuretic peptide, *RA* right atrial pressure, *PCWP* pulmonary capillary wedge pressure, *mPAP* mean pulmonary artery pressure, *CI* cardiac index, *PVR* pulmonary vascular resistance, *SvO*_*2*_ mixed venous oxygen saturation, *IPAH* idiopathic pulmonary arterial hypertension.

### Baseline copeptin levels are elevated in patients with PAH

**Figure 1:**
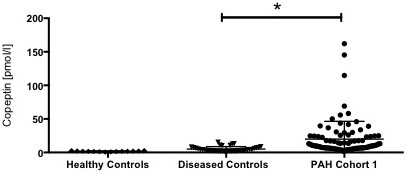
**Copeptin levels in controls and patients with PAH from cohort 1 at baseline.** Data are shown as median (IQR). Differences between the groups were assessed using Kruskal–Wallis one-way analysis of variance. * indicates p < 0.05.

The mean copeptin level of PAH patients was four to five times higher compared to diseased controls and ten times higher compared to healthy controls. Median copeptin levels in the two PAH cohorts were 20.1 pmol/l (IQR 7–25) and 24.5 pmol/l (IQR 8–37) respectively. There were no statistical significant differences between patients with IPAH or APAH (Additional file 1: Table S4). The median copeptin level in the diseased control group was 5.1 pmol/l (IQR 2–7) and 1.8 pmol/l (IQR 1.4-2.2) in the healthy controls. Both statistically different from the two PAH cohorts (p < 0.001) (Table 2, Figure 1).

### NT-proBNP and copeptin ROC analysis

Receiver operating characteristic of NT-proBNP and copeptin to predict mortality after 3 years showed that the area under the curve (AUC) for NT-proBNP was 0.71 (CI 0.59-0.83, 0.02) and 0.77 (CI 0.64 -0.86, p = 0.001) for copeptin. The best cutoff level to predict survival for NT-proBNP was 1288 pg/ml (sensitivity of 71%, specificity of 63%) and 13.2pmol/l (sensitivity of 70%, specificity of 78%) for copeptin respectively (Additional file 2: Figure S1).

### Baseline copeptin levels correlate with parameters of disease severity in PAH

Patients presenting with baseline copeptin levels above the copeptin cutoff of 13.2 pmol/l presented with higher NYHA class (p = 0.01), increased creatinine levels (p = 0.04), decreased eGFR-CKD-EPI (p = 0.03) and higher NT-proBNP levels (p < 0.001). There were no significant differences in both groups regarding demographic data, 6MWD, S-Na concentration, presence of edema, or hemodynamic parameters (Table 3).

**Table 3:** Baseline characteristics in relation to copeptin levels

	**Copeptin <13.2 pmol/l (n = 54)**	**Copeptin >13.2 pmol/l (n = 38)**	**p-value**
Age (years)	55.2 (45–67)	57.4 (50–71)	0.63
Gender (%female)	72	52	0.49
6MWD [m]	344 (252–489)	303 (202–406)	0.43
NYHA class [%]			0.012
II	32	13	
III	67	76	
IV	2	11	
Edema (%)	35	45	0.38
S-Na [mmol/l]	139 (138–143)	139 (136–141)	0.46
Creatinine [μg/l]	87.2 (61–94)	91.5 (71–105)	0.04
eGFR (CKD-EPI) [ml/min]	87 (74–99)	78 (60–93)	0.03
NT-proBNP [pg/ml]	1562 (351–18)	3057 (1678–4531)	<0.001
RA [mmHg]	5.3 (2–8)	7.8 (2–12)	0.43
PCWP [mmHg]	8.8 (6–12)	8.1 (6–10)	0.45
mPAP [mmHg]	45 (32–67)	46.2 (39–54)	0.56
CI [ml/min/m^2^]	2.5 (1.9-2.9)	2.2 (1.9-2.9)	0.12
PVR [dyn · sec · cm^-5^]	777 (419–1032)	814 (541–980)	0.26
SvO_2_ [%]	65 (58–73)	60 (56–67)	0.06

PAH patients from cohort 1 were dichotomized according their copeptin levels (<13.2 pmol/l or >13.2 pmol/l) and demographic, clinical, biochemical and hemodynamic variables from both groups were compared. Differences were calculated by using Mann–Whitney U-test for continuous and χ^2^ -test for categorical variables.

Except for sex, NYHA class and edema (%), data are shown as median (interquartile range, IQR).

Definition of *abbreviations*: *6MWD* 6 minute walking distance, *NYHA* New York Heart Association, *S-Na* Serum Sodium concentration, *eGFR (CKD-EPI)* estimated glomerular filtration calculated according the Chronic Kidney Disease Epidemiology Collaboration formula, *NT-proBNP* N-terminal prohormone of brain natriuretic peptide, *RA* right atrial pressure, *PCWP* pulmonary capillary wedge pressure, *mPAP* mean pulmonary artery pressure, *CI* cardiac index, *PVR* pulmonary vascular resistance, *SvO*_*2*_ mixed venous oxygen saturation.

There was a significant correlation between NYHA class and copeptin levels (r = 0.46, p < 0.01) (Table 4). Patients presenting in NYHA class II had lower levels of copeptin compared to patients presenting in NYHA III or IV, respectively; 8.2 pmol/l (IQR 4–14), vs. 20.3 pmol/l (IQR 14–28), vs. 35.9 pmol/l, (IQR 26–49) (p < 0.001) (Additional file 3: Figure S2).

**Table 4:** Correlations of serum copeptin levels and serum NT-proBNP levels with demographic, clinical, biochemical and hemodynamic parameters

	**Copeptin**		**NT-proBNP**	
Parameter	rho	p	rho	p
Age (years)	0.21	0.05	0.18	0.21
Gender (female%)	0.13	0.09	-0.85	0.49
**6MWD [m]**	**-0.26**	**0.04**	**-0.51**	**0.01**
**NYHA class**	**0.46**	**0.01**	**0.29**	**0.04**
Edema	0.06	0.48	0.168	0.18
S-Na [mmol/l]	0.19	0.45	-0.03	0.83
**Creatinine [μg/l]**	**0.39**	**0.01**	**0.32**	**0.01**
**eGFR (CKD-EPI) [ml/min]**	**-0.32**	**0.01**	**-0.40**	**0.03**
**NT-proBNP [pg/ml]**	**0.49**	**0.01**	-	-
Copeptin [pmol/l]	-	-	**0.49**	**0.01**
RA [mmHg]	0.19	0.06	**0.41**	**0.01**
PCWP	0.09	0.41	0.23	0.14
mPAP [mmHg]	0.06	0.91	0.23	0.07
CI [ml/min/m^2^]	-0.14	0.18	**-0.51**	**0.01**
PVR [dyn · sec · cm^-5^]	0.06	0.57	**0.35**	**0.01**
SvO_2_ [%]	-0.22	0.07	**-0.60**	**0.01**

Data are from the retrospective cohort (cohort 1).

The relations between copeptin and NT-proBNP levels and baseline variables were assessed by Spearman rank correlation coefficients.

Significant correlations are highlighted in boldface.

Definition of *abbreviations*: *6MWD* 6 minute walking distance, *NYHA* New York Heart Association, *S-Na* Serum Sodium concentration, *eGFR (CKD-EPI)* estimated glomerular filtration calculated according the Chronic Kidney Disease Epidemiology Collaboration formula, *NT-proBNP* N-terminal prohormone of brain natriuretic peptide, *RA* right atrial pressure, *PCWP* pulmonary capillary wedge pressure, *mPAP* mean pulmonary artery pressure, *CI* cardiac index, *PVR* pulmonary vascular resistance, *SvO*_2_ mixed venous oxygen saturation.

Copeptin levels were negatively correlated with 6MWD (r = -0.26, p = 0.04) and eGFR-CKD-EPI (r = -0.32, p = 0.01). Circulating copeptin levels at baseline were not associated with serum sodium concentration frequency of edema (r = 0.05, p = 0.39; r = 0.09, p = 0.45) but strongly related to NT-proBNP levels (r = 0.59, p < 0.001). NT-proBNP levels were related to 6MWD (r = -0.51, p < 0.01), NYHA class (r = 0.29, p = 0.04), creatinine (r = 0.32, p = 0.01), eGFR-CKD-EPI (r = -0.40, p < 0.03), right atrial pressure (RA) (r = 0.41, p < 0.01), cardiac index (CI) (r = -0.51, p < 0.01), pulmonary vascular resistance (PVR) (r = 0.35, p = 0.01) and mixed venous oxygen saturation (Sv0_2_) (r = -0.60, p < 0.01) (Table 4).

### Baseline copeptin levels are associated with outcome

Univariate Cox Regression analysis showed that baseline copeptin levels were a significant prognostic indicator of poor outcome (HR 1.9, CI 1.4-2.4, p < 0.001). Multiple forward Cox Regression analysis showed that copeptin levels remained a significant predictor of mortality when tested against NT-proBNP, serum sodium concentration (S-Na) and eGFR CKD-EPI (HR 1.4; 95%, CI 1.1-2.0, p = 0.04) (Table 1).

**Figure 2:**
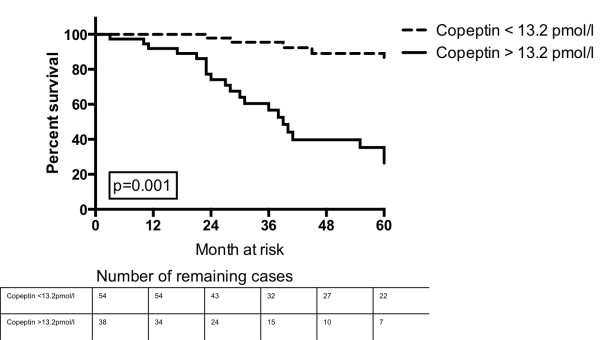
Probability of survival according to baseline copeptin levels above or below the ROC derived cutoff of 13.2 pmol/l.

Kaplan Meier survival analysis showed that patients with a ROC derived cutoff copeptin level <13.2 pmol/l at baseline had a significantly better survival compared to patients with a copeptin level > 13.2 pmol/l (Figure 2). The estimated median survival of patients with a copeptin level <13.2 pmol/l was 103 months (IQR 132–84), compared to 52 (IQR 84 – 24) months in patients with a copeptin level >13.2 pmol/l (p < 0.001).

### Baseline copeptin levels add prognostic information to baseline NT-proBNP levels

**Figure 3:**
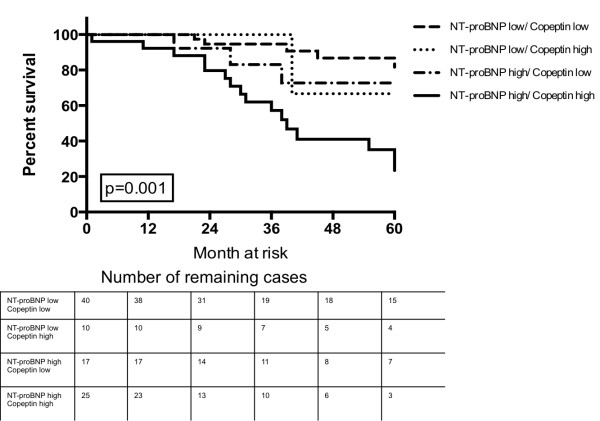
Probability of survival according to baseline NT-proBNP and copeptin levels above or below the ROC derived cutoffs.

Kaplan Meier survival analysis showed that patients with a ROC derived cutoff NT-proBNP level <1288 pg/ml and a copeptin level <13.2 pmol/l at baseline had significant better chances of survival compared to patients with an elevation above the cutoff in both biomarkers. The estimated median survival of patients with a NT-proBNP and copeptin below the ROC derived cutoffs was 114 month (IQR 128–89), compared to 47 month (IQR 61–33) in patients with an elevation in both biomarkers (p < 0.001) (Figure 3).

### Copeptin levels decrease after initiation of PAH targeted treatment

After initiation of PAH targeted therapy, there was significant improvement in all measured pulmonary hemodynamic variables. Changes in NYHA class and 6MWD did not reach clinical significance. Copeptin levels decreased significantly from 24.5 pmol/l at baseline to 15.8 pmol/l after 3 to 6 months of treatment (p = 0.01) (Additional file 1: Table S1).

Changes in copeptin levels did not correlate with changes in pulmonary or systemic hemodynamic parameters. There was no correlation between changes in 6MWD, eGFR-CKD-EPI, S-Na and changes in copeptin, however the association between changes in NT-proBNP and changes in copeptin was significant (r = 0.53, p = 0.01) (Additional file 1: Table S3).

## Discussion

### Copeptin as a New biomarker in PAH

Copeptin has emerged as a promising surrogate target for measurement of vasopressin concentration and also seems useful in cardiovascular disease [21,22].

Patients with PAH presented with markedly elevated copeptin levels compared to patients without a diagnosis of PAH. Increased copeptin levels were associated with a higher risk of death and an independent predictor of poor outcome.

Copeptin levels were significantly correlated with NYHA class, 6MWD and kidney function, all of which being indicators of more severe disease and poor outcome in PAH patients [3,23-25].

As reported in patients with left ventricular heart failure [14] PAH patients showed a strong association between copeptin and NT-proBNP levels at baseline and after initiation of treatment. It is known (and confirmed in this study) that NT-proBNP shows clear associations with pulmonary hemodynamics and it is released mainly by the right atrium in PAH patients [16,26]. Given the independence of copeptin from pulmonary hemodynamics and the consistent correlation with NT-proBNP at baseline and after beginning of PAH targeted therapy, copeptin levels might reflect neurohumoral activation due to altered right ventricular function. Therefore elevated copeptin levels at baseline and during follow up could add important prognostic information that is not captured by right heart catheterization or NT-proBNP levels alone. Multimarker strategies for risk stratification are increasingly used in patients with coronary syndrome [27] and left heart failure [15,28].

Copeptin was shown to provide additional information to troponin in the triage of chest pain patients and to improve the diagnostic performance of NT-proBNP in predicting risk of all-cause mortality in patients with chronic heart failure [22,28].

Given the limitations of a NT-proBNP when it comes to predicting individual outcomes in PAH, the combination of copeptin with NT-proBNP might help to distinguish a patient population with particular high risk for fatal outcome.

### Copeptin and indicators of neurohumoral activation in PAH

Peripheral edema and ascites are common presentations in patients with advanced PAH and indicators of increased neurohumoral activation. The role of neurohumoral mediators in PAH is of great interest since the pathophysiology of volume overload in right heart failure is not well understood. There is cumulating evidence for increased neurohumoral activation in PAH. It was shown that PAH patients have elevated levels of norepinephrine [16], renin, aldosterone, elevated plasma volume and hyponatremia in advanced disease [17-20]. In this report we show that copeptin levels are also strikingly elevated in PAH, providing further evidence for increased neurohumoral activation in those patients.

Unlike previously reported in patients with chronic left heart failure [14] we found no significant correlation between circulating copeptin levels and other indicators of increased neurohumoral activation (e.g. decreased serum sodium concentrations or the presence of edema). One explanation could be a lack of statistical power in this study due to relatively low sample size compared to studies in patients with left heart failure. However, a recent clinical study in acute heart failure patients did also find no association of right ventricular preload, copeptin levels, serum sodium concentration and the presence of edema [29].

Decreased serum sodium concentration and peripheral edema in chronic heart failure are associated with chronically elevated levels of neurohumoral mediators, and are usually seen in late disease stages when decompensation occurs. Activation of the AVP system, measured by copeptin levels, in contrast, appears to occur early in the course of the disease.

Taken together, these data might suggest that activation of the AVP system - measured by copeptin levels - is not the primary cause of volume overload and hyponatremia among patients with right heart strain, but might reflect an early indication of neurohumoral stimulation. But the exact relationship between neurohumoral hormones and other mediators of sodium and fluid homeostasis in the setting of right heart strain needs further investigation.

### Vasopressin in right ventricular failure

Vasopressin release is triggered following reduced cardiac output and activation of baroreceptors in the carotid sinus [30] leading to systemic vasoconstriction and renal water retention [5].

The lack of association of copeptin with cardiac output and mixed venous oxygen saturation, but the strong correlation with functional parameters like 6MWD and NYHA class provides evidence that activation of the AVP-system reflects an interplay of a variety of different factors, rather than cardiac function alone, that determine overall cardiovascular performance in PAH patients.

The decrease of copeptin levels after initiation of PAH targeted therapy suggests that longitudinal copeptin measurements might provide important information about cardiovascular stress and response to treatment.

Excessive neurohumoral activation contributes to the development of symptoms of heart failure by altering vascular resistance, blood volume and cardiac contractility [31]. In addition, vasopressin is involved in cardiac remodeling via its V_1_receptor on cardiomyocytes, leading to increased protein-synthesis, cardiac hypertrophy, decreased contractility and development of myocardial fibrosis [32,33]. These observations, although mostly derived from the left ventricle, raise the possibility that elevated vasopressin levels in PAH patients might also play a role in right ventricular remodeling [34].

### Limitations of the study

This was a single-center, mostly retrospective study with a limited sample size, especially in the prospective cohort. Given the descriptive nature of the current study, this manuscript does not address the causes or physiologic mechanisms for elevated copeptin levels in patients with PAH.

## Conclusions

Circulating copeptin levels are elevated in patients with PAH and have the potential to become useful biomarkers in the assessment of PAH patients adding independent information to the clinical, biochemical and hemodynamic assessment.

## Abbreviations

6MWD: Six minute walking distance; CI: Cardiac index; eGFR: Estimated glomerular filtration rate; eGFR-CKD-EPI: Estimated glomerular filtration calculated according the chronic Kidney disease epidemiology collaboration formula; mPAP: Mean Pulmonary Arterial pressure; NT-proBNP: N-terminal prohormone of brain natriuretic peptide; NYHA: New York Heart Association; PAH: Pulmonary arterial hypertension; PCWP: Pulmonary capillary wedge pressure; PVR: Pulmonary vascular resistance; RA: Right atrial pressure; S-Na: Serum Sodium Concentration; SvO2: Mixed venous oxygen saturation.

## Competing interest

None of the authors has any conflict of interest with the data published in this manuscript. NPN: received funding from Deutsche Forschungsgemeinschaft (DFG). RL: has nothing to disclose. HG: is member of the advisory board of Roche pharmaceuticals and Boehringer Pharmaceuticals, and received lecture fees from Pfizer pharmaceuticals. KMO: received lecturing and traveling fees from Pfizer and Actelion. KB: has nothing to disclose. TW: is member of the advisory board of Brahms pharmaceuticals and received lecturing fees from Brahms and Bio Merieux pharmaceuticals. MMH: received consultant and lecture fees from Actelion, Bayer, GSK, Lilly, Pfizer, Novartis.

## Authors’ contribution

NPN contributed to study design, acquisition, analysis and interpretation of the data and wrote the manuscript. RL contributed to copeptin, NT-pro-BNP measurements. He contributed to study design and interpretation of the data. HG contributed to data and sample acquisition and edited the manuscript. KMO contributed to data and sample acquisition and edited the manuscript. KB contributed to copeptin, NT-pro-BNP measurements. TW supervised the project. MMH designed the study, interpreted the data and wrote the paper. NPN had full access to all the data in the study and that he takes responsibility for the integrity of the data and the accuracy of the data analysis. All authors read and approved the final manuscript.

## Supplementary Material

Additional file 1: Table S1 Changes in functional, biochemical and biochemical variables, from baseline to 3-6 month follow up. **Table S2.** PAH medication of cohort 2. **Table S3.** Relationship between changes in serum copeptin levels and changes in clinical, biochemical and hemodynamic parameters after initiation of PAH treatment. **Table S4.** Copeptin levels in PAH-Subgroups from PAH cohort 1. **Table S5.** Adjusted risk of death in relation to copeptin. **Table S6.** Risk of death in relation to significant variables from the single parameter cox regression analysis. Click here for file

Additional file 2: Figure S1 Receiver Operating Characteristic Curve Analyses and Area Under The Curve Statistics Relating NT-proBNP and Copeptin Levels To 3-year Outcome In Cohort 1. Click here for file

Additional file 3: Figure S2 Copeptin Levels According To NYHA Class From Cohort 1. Data are shown as median (IQR). Differences between the groups were assessed using Kruskal-Wallis one-way analysis of variance. *indicates p < 0.05. Click here for file
