# Supplementary material for: Circulating levels of copeptin predict outcome in patients with pulmonary arterial hypertension
Source: Respir Res. 2013 Nov 19;14(1):130. doi: 10.1186/1465-9921-14-130 (PMC4176098; doi:10.1186/1465-9921-14-130)
Supplement: Additional file 1: Table S1 — Changes in functional, biochemical and biochemical variables, from baseline to 3-6 month follow up. Table S2. PAH medication of cohort 2. Table S3. Relationship between changes in serum copeptin levels and changes in clinical, biochemical and hemodynamic parameters after initiation of PAH treatment. Table S4. Copeptin levels in PAH-Subgroups from PAH cohort 1. Table S5. Adjusted risk of death in relation to copeptin. Table S6. Risk of death in relation to significant variables from the single parameter cox regression analysis. [file 1465-9921-14-130-S1.doc]

Circulating Levels of Copeptin Predict Outcome in Patients with Pulmonary Arterial Hypertension

**Data Supplement**

**Figures/tables: 2/6**

**Additional file 2: Figure S1: Copeptin Levels According To NYHA Class From Cohort 1**

Data are shown as median (IQR)

Differences between the groups were assessed using Kruskal-Wallis one-way analysis of variance.

* indicates p<0.05.

**Additional file 2: Figure S2: Receiver Operating Characteristic Curve Analyses and Area Under The Curve Statistics Relating NT-proBNP and Copeptin Levels To 3-year Outcome In Cohort 1.**

**Additional file 1: Table S1: Changes In Functional, Biochemical And Biochemical Variables, From Baseline To 3-6 Month Follow Up**

Data are from the prospective cohort (cohort 2).

Data are shown as median (interquartile range).

Changes from baseline to 3–6 months follow-up were assessed by Wilcoxon signed rank test.

Significant changes from baseline to follow up are highlighted in bold.

|  | Baseline | Follow-up | p-value |
| --- | --- | --- | --- |
| 6MWD [m] | 352 (295-413) | 367 (305-445) | 0.19 |
| S-Na [mmol/l] | 143 (139-145) | 143 (139-145) | 0.98 |
| eGFR (CKD-EPI) [ml/min] | 102.6 (78-125) | 96.9 (81-131) | 0.06 |
| **NT-proBNP [pg/ml]** | **2063 (990-2541)** | **1256 (530-1812)** | **0.02** |
| **Copeptin [pmol/l]** | **24.5 (10-33)** | **15.8 (5-21)** | **0.01** |
| **RA [mmHg]** | **7.7 (3-12)** | **4.8 (1-7)** | **0.04** |
| **mPAP [mmHg]** | **50 (43-56)** | **41 (27-51)** | **0.02** |
| **CI [ml/min/m^2^]** | **1.9 (1.4-2.5)** | **2.4 (1.9-2.8)** | **0.01** |
| **PVR [[dyn·sec·cm^‑5^]** | **1004 (670-1325)** | **729 (470-1039)** | **0.02** |
| **SvO2 [%]** | **61.2 (56-68)** | **66.5 (63-72)** | **0.03** |

Definition of abbreviations: 6MWD= 6 minute walking distance; S-Na= Serum Sodium concentration; eGFR (CKD-EPI)= estimated glomerular filtration calculated according the Chronic Kidney Disease Epidemiology Collaboration formula; NT-proBNP= N-terminal prohormone of brain natriuretic peptide; RA= right atrial pressure; mPAP= mean pulmonary artery pressure; CI= cardiac index; PVR= pulmonary vascular resistance; SvO_2_= mixed venous oxygen saturation.

**Additional file 1: Table S2: PAH Medication Of Cohort 2**

Data are shown as percent of patients on indicated therapy.

| PAH Medication [%] |  |
| --- | --- |
| PDE-Inhibitor | 51 |
| ERA | 40 |
| Combination therapy | 9 |

**Additional file 1: Table S3: Relationship Between Changes In Serum Copeptin Levels And Changes In Clinical, Biochemical And Hemodynamic Parameters After Initiation Of PAH Treatment.**

Data are from the prospective cohort (cohort 2).

Changes (Delta) in Copeptin in relation to changes (Delta) in baseline variables over time were assessed by Spearman rank correlation coefficients.

Significant correlations are highlighted in bold.

| Change in Copeptin (follow up – baseline) | | |
| --- | --- | --- |
|  | Pearson | p |
| Changes in Variables  (follow up – baseline) |  |  |
| 6MWD [m] | -0.35 | 0.42 |
|  |  |  |
| S-Na [mmol/l] | 0.05 | 0.54 |
| eGFR (CKD-EPI) [ml/min] | -0.14 | 0.69 |
| **NT-proBNP [pg/ml]** | **0.53** | **0.03** |
|  |  |  |
| RA [mmHg] | -0.42 | 0.89 |
| mPAP [mmHg] | -0.28 | 0.31 |
| CI [ml/min/m^2^] | 0.04 | 0.89 |
| PVR [dyn·sec·cm^‑5^] | -0.34 | 0.56 |
| SvO_2_ [%] | 0.02 | 0.97 |

Definition of abbreviations: 6MWD= 6 minute walking distance; S-Na= Serum Sodium concentration; eGFR (CKD-EPI)= estimated glomerular filtration calculated according the Chronic Kidney Disease Epidemiology Collaboration formula; NT-proBNP= N-terminal prohormone of brain natriuretic peptide; RA= right atrial pressure; mPAP= mean pulmonary artery pressure; CI= cardiac index; PVR= pulmonary vascular resistance; SvO_2_= mixed venous oxygen saturation.

**Additional file 1: Table S4: Copeptin Levels in PAH-Subgroups From PAH Cohort 1.**

Shown are baseline copeptin levels from PAH cohort 1.

| PAH Group | Copeptin (median IQR) | p-value |
| --- | --- | --- |
| IPAH [n=59] | 18.4 (8-21) | ns |
| APAH [n=33] | 24.5 (10-33) |
| CTD [n=23] | 26.4 (14-31) |  |
| CHD [10] | 19.2 (10-23) |  |

Definition of abbreviations: APAH= associated pulmonary arterial hypertension; CTD= connective tissue disease; CHD= congenital heart disease; ns= not significant.

**Additional file 1: Table S5: Adjusted Risk Of Death In Relation To Copeptin.**

Data are from the retrospective cohort (cohort 1).

Estimated HRs, 95% CIs, and p values were calculated by Cox regression analyses.

Supplement Table 5:

Adjusted Models for Copeptin

| Parameter | Single Model | p-value |
| --- | --- | --- |
| **Unadjusted** | HR (CI) |  |
| Copeptin (per 5pmol/l🡹) | 1.9 (1.4 – 2.4) | 0.001 |
|  |  |  |
| **Adjusted for age** |  |  |
| Copeptin (per 5pmol/l🡹) | 1.6 (1.2 – 2.5) | 0.001 |
|  |  |  |
| **Adjusted for gender** |  |  |
| Copeptin (per 5pmol/l🡹) | 1.7 (1.4 – 2.7) | 0.002 |

**Additional file 1: Table S6: Risk Of Death In Relation To Significant Variables From The Single Parameter Cox Regression Analysis.**

Data are from the retrospective cohort (cohort 1).

Estimated HRs, 95% CIs, and p values were calculated by multiple stepwise forward Cox regression analyses.

| 6MWD (30 m🡻) | 1.1 (1.0 – 1.3) | 0.013 |
| --- | --- | --- |
| NYHA | 0.8 (0.6-1.1) | 0.284 |
| Copeptin (per 5pmol/l🡹) | 1.3 (1.1 -1.4) | 0.010 |

Definition of abbreviations: 6MWD= 6 minute walking distance; NYHA= New York Heart Association.
