# Supplementary figures and images for: Circulating levels of copeptin predict outcome in patients with pulmonary arterial hypertension
Source: Respir Res. 2013 Nov 19;14(1):130. doi: 10.1186/1465-9921-14-130 (PMC4176098; doi:10.1186/1465-9921-14-130)

## Slide 1
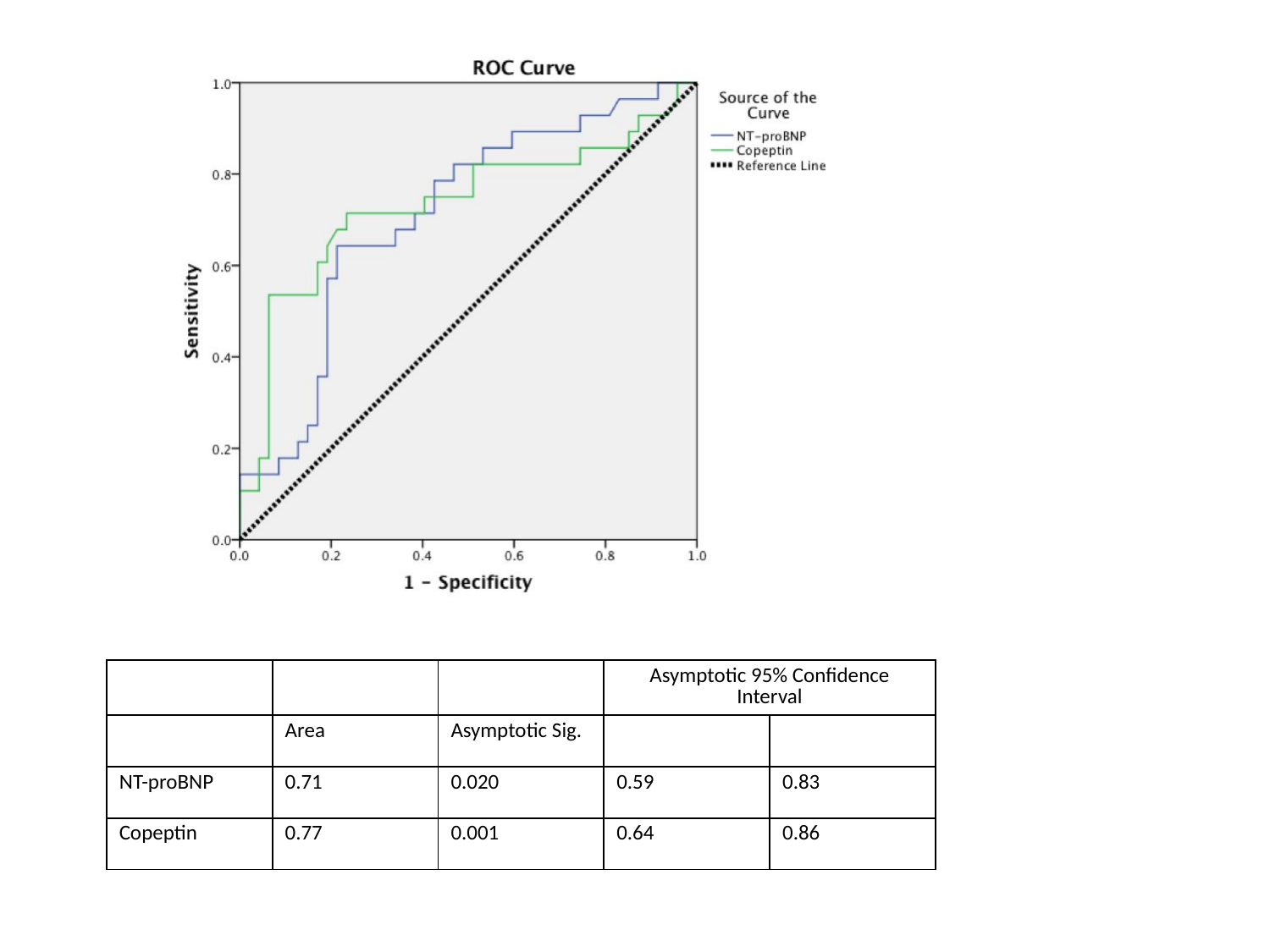

| | | | Asymptotic 95% Confidence Interval | |
| --- | --- | --- | --- | --- |
| | Area | Asymptotic Sig. | | |
| NT-proBNP | 0.71 | 0.020 | 0.59 | 0.83 |
| Copeptin | 0.77 | 0.001 | 0.64 | 0.86 |

Supplement: Additional file 2: Figure S1 — Receiver Operating Characteristic Curve Analyses and Area Under The Curve Statistics Relating NT-proBNP and Copeptin Levels To 3-year Outcome In Cohort 1. [file 1465-9921-14-130-S2.pptx]

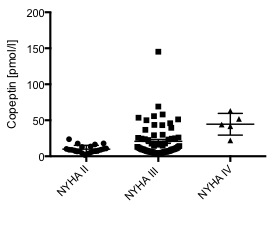

Supplement: Additional file 3: Figure S2 — Copeptin Levels According To NYHA Class From Cohort 1. Data are shown as median (IQR). Differences between the groups were assessed using Kruskal-Wallis one-way analysis of variance. *indicates p < 0.05. [file 1465-9921-14-130-S3.jpeg]
